# Effect of heart failure on catecholamine granule morphology and storage in chromaffin cells

**DOI:** 10.1530/JOE-16-0146

**Published:** 2016-09-01

**Authors:** Sushil K Mahata, Hong Zheng, Sumana Mahata, Xuefei Liu, Kaushik P Patel

**Affiliations:** 1 VA San Diego Healthcare System Metabolic Physiology & Ultrastructural Biology Lab.Department of Medicine, University of California at San Diego, La Jolla, CA, USA; 2 Department of Cellular and Integrative PhysiologyUniversity of Nebraska Medical Center, Omaha, NE, USA; 3 Caltech Division of BiologyCalifornia Institute of Technology, Pasadena, CA, USA

**Keywords:** catecholamines, chromaffin cells, heart failure, sympathetic activation

## Abstract

One of the key mechanisms involved in sympathoexcitation in chronic heart failure (HF) is the activation of the adrenal glands. Impact of the elevated catecholamines on the hemodynamic parameters has been previously demonstrated. However, studies linking the structural effects of such overactivation with secretory performance and cell metabolism in the adrenomedullary chromaffin cells *in vivo* have not been previously reported. In this study, HF was induced in male Sprague-Dawley rats by ligation of the left coronary artery. Five weeks after surgery, cardiac function was assessed by ventricular hemodynamics. HF rats showed increased adrenal weight and adrenal catecholamine levels (norepinephrine, epinephrine and dopamine) compared with sham-operated rats. Rats with HF demonstrated increased small synaptic and dense core vesicle in splanchnic–adrenal synapses indicating trans-synaptic activation of catecholamine biosynthetic enzymes, increased endoplasmic reticulum and Golgi lumen width to meet the demand of increased catecholamine synthesis and release, and more mitochondria with dilated cristae and glycogen to accommodate for the increased energy demand for the increased biogenesis and exocytosis of catecholamines from the adrenal medulla. These findings suggest that increased trans-synaptic activation of the chromaffin cells within the adrenal medulla may lead to increased catecholamines in the circulation which in turn contributes to the enhanced neurohumoral drive, providing a unique mechanistic insight for enhanced catecholamine levels in plasma commonly observed in chronic HF condition.

## Introduction

Enhanced neurohumoral drive is a major risk factor that influences the progression of chronic heart failure (HF) and mortality in patients and the experimental models (Packer 1988, Zucker *et al*. 1995, Patel 1997). During HF, enhanced levels of catecholamines resulting from activation of the sympathetic nervous system ensure an increase in cardiac function, to achieve an adaptation of the cardiac output to the systemic needs. However, long-term stimulation of catecholamines becomes harmful, contributing to the progression of HF. Although most therapeutic pharmaceutical strategies target the peripheral symptoms of the disease, they may not influence the enhanced sympathetic nerve activity. Indeed, various studies have detailed the role of cardiac and hemodynamic mechanisms involved in the elevated sympathetic drive in HF (Packer 1988, Ferguson *et al*. 1990, Brändle *et al*. 1996, Patel 1997). The role of the adrenal gland in the development of sympathetic overactivation and progression of HF is less well known.

Circulating catecholamines, comprising dopamine (DA), norepinephrine (NE) and epinephrine (EPI), are primarily synthesized and released from the chromaffin cells of the adrenal medulla (Goldstein *et al*. 2003). Under physiological (nonstress) conditions, the adrenal medulla secretes ~80% of EPI and ~20% of NE (Tank & Lee Wong 2015). While EPI is secreted exclusively from the adrenal medulla, the major source of circulating NE is from sympathetic nerve endings (Goldstein *et al*. 2003). Plasma DA comes from the adrenal medulla, sympathetic nerve endings and the brain. Resting levels of plasma catecholamines range from 25 to 70pg/mL for DA, 200 to 300pg/mL for NE and 30 to 70pg/mL for EPI (Tank & Lee Wong 2015). Although plasma DA concentration is similar to those of EPI, circulating DA does not act as a hormone because of its much lower potency. Plasma concentrations of NE and EPI increase dramatically in response to physical, psychological or environmental stress, exercise, exposure to cold or low oxygen tension or fear and alarm to counteract with the stress (Tank & Lee Wong 2015). This biological response of catecholamines is considered as an adaptive response.

The contribution of adrenal catecholamines to the progression of HF has been documented in a few experimental models (Lymperopoulos *et al*. 2010, Schneider *et al*. 2011). Adrenal gland hypertrophy has been reported in rats after experimental myocardial infarction (Schneider *et al*. 2011). Mice lacking α_2C_-adrenoceptors which control EPI secretion in adrenal chromaffin cells show rapid deterioration of cardiac function and raised mortality after transverse aortic constriction (de Lucia *et al*. 2014). Interestingly, reduction in sympathetic activity via adrenal-targeted G-protein-coupled receptor kinase 2 (GRK2) gene deletion attenuates HF progression and improves cardiac function after myocardial infarction (Lymperopoulos *et al*. 2010). Therapeutic strategies such as β-blocker and exercise training have been reported to improve outcome in HF, likely by lowering HF-dependent sympathetic nervous system hyperactivity, and at least in part, via reduction in adrenal catecholamine hypersecretion (Rengo *et al*. 2010, de Lucia *et al*. 2014).

Elevated levels of circulating catecholamines have been reported in both patients with HF and animals with experimental HF (Cohn *et al*. 1984, Kleiber *et al*. 2008). Under the stress condition, the increase in plasma EPI is derived almost completely from the adrenal medulla, whereas about 70% plasma NE is derived from sympathetic nerves (Kvetnansky *et al*. 1979). Exaggerated sympathetic nerve activation is a major risk factor that influences the progression of HF and mortality in patients. High levels of plasma NE in patients with HF have been known to have very poor prognosis (Cohn *et al*. 1984). Although most therapeutic pharmaceutical strategies target the peripheral symptoms of the disease, they may not influence the overall enhanced sympathetic activation. Reducing the adrenergic drive is important, as HF patients with lower levels of plasma NE are given a better prognosis (Cohn *et al*. 1984). However, the adrenal medullary contribution and alterations for the origin of catecholamines in the circulation during HF condition are not fully explored to date. Thus, we hypothesized that the structural effects of secretory performance and cell metabolism in the adrenal medullary chromaffin cells may lead to increased catecholamines in the circulation which in turn contributes to the enhanced neurohumoral drive commonly observed in the HF condition.

## Materials and methods

### Study approval

All the procedures on animals in this study were approved by the University of Nebraska Medical Center Institutional Animal Care and Use Committee. The experiments were conducted according to the American Physiological Society guiding principles for research involving animals and human beings and the NIH guide for the care and use of laboratory animals.

### Induction of heart failure

Male Sprague-Dawley rats weighing 200–220g were obtained from Sasco Breeding Laboratories (Omaha, NE, USA) and were randomly assigned to a sham-operated group and an HF group. HF was produced by coronary artery ligation, as described previously (Zhang *et al*. 2001, Kleiber *et al*. 2008). Each rat was caged individually in an environment with ambient temperature maintained at 22°C and humidity at 30–40%. Laboratory chow and tap water were available *ad libitum*.

The degree of left ventricular dysfunction and HF were determined using both hemodynamic and anatomic criteria. Echocardiograms were performed at 5 weeks after surgery. Left ventricular end-diastolic pressure (LVEDP) was measured using a Mikro-Tip catheter (Millar Instruments, Houston, TX, USA) at the time of the terminal experiment. To measure infarct size, the heart was dissected and the atria and right ventricle were removed. A digital image of the left ventricle was captured using a digital camera. The percentage of infarct area to total left ventricle area was quantified using SigmaScan Pro (Aspire Software International, Ashburn, VA, USA). Rats with both LVEDP >15mmHg and infarct size >30% of total left ventricular wall were considered to be in HF. Sham-operated rats were treated similarly as the HF rats except that their coronary arteries were not ligated.

### Adrenal catecholamine measurements

The adrenal glands were harvested from each animal immediately after killing. These samples were weighed, homogenized in cold perchloric acid containing ethylenediaminetetraacetic acid and centrifuged at 4°C for 15min at 15,000***g***. The supernatants were stored at −70°C until they were assayed for catecholamines. DA, NE and EPI concentrations were assayed using alumina extraction followed by separation and analysis with HPLC and electrochemical detection (Bioanalytical Systems, West Lafayette, IN, USA). Dihydroxybenzylamine (Sigma) was used as an internal standard in each tissue sample (Kline *et al*. 1986).

### Urinary norepinephrine excretion measurement

Urinary NE excretion was measured as an index of overall sympathetic activation. Five weeks after surgery, rats from all groups were placed in metabolic cages and urine was collected for 24h. Urinary NE concentration of thawed samples was measured using a commercially available ELISA kit (Labor Diagnostika Nord, Nordhorn, Germany), following the manufacturer’s instructions.

### Real-time RT-PCR and Western blot measurement of catecholamine biosynthetic enzymes in the adrenal gland

The half adrenal gland was placed in 500μL Tri-Reagent (Molecular Research Center Inc, OH, USA), followed by extraction of mRNA. The other half of adrenal gland was placed in 150μL RIPA lysis buffer to extract the protein. Real-time RT-PCR measurements were made with the iCycler iQ Multicolor Real-Time Detection System (Bio-Rad). The reaction mixture consisted of SYBR Green Supermix (Bio-Rad), 300nM sense and antisense primers, and the cDNA template of interest (for primer sequences see Table 1). Relative expression of tyrosine hydroxylase (TH, the rate limiting enzyme in catecholamine biosynthesis, which converts tyrosine to l-Dopa), dopamine-β-hydroxylase (DβH, which converts dopamine to NE) and phenylethanolamine N-methyltransferase (PNMT, which converts NE to EPI) mRNA was calculated with the Pfaffl equation, which relates the expression of the target gene to the expression of reference gene: glyceraldehyde 3-phosphate dehydrogenase (GAPDH).

**Table 1:** Primer sequences used for quantitative real-time PCR analysis.

**Gene**	**Primer sequence**
TH	Sense: CTGGGTGCACTTGTCTGTGCAGT
	Antisense: CAGTACACCGTGGAGAG
DβH	Sense: CACCACATCATCATGTATGAGG
	Antisense: CCTGTCTGTGCAGTAGCCAG
PNMT	Sense: TACCTCCGCAACAACTACGC
	Antisense: AAGGCTCCTGGTTCCTCTCG
GAPDH	Sense: TGACAACTCCCTCAAGATTTGTCA
	Antisense: GGCATGGACTGTGGTCATGA

The protein samples were loaded onto an SDS-PAGE gel and subjected to electrophoresis. The fractionated proteins on the gel were transferred to a polyvinylidene difluoride membrane (Millipore). The membrane was probed with primary antibody (rabbit anti-TH (1:500), DβH (1:500) and PNMT (1:1000) (Abcam) or mouse anti-β-actin (1:1000, Santa Cruz)) overnight, and then probed with secondary antibody (peroxidase-conjugated anti-rabbit or goat IgG, 1:5000, Pierce). An enhanced chemiluminescence substrate (Pierce) was applied to the membrane, followed by an exposure within an UVP system (UVP BioImaging, CA, USA) for visualization. Kodak 1D software (Kodak) was used to quantify the signal. The expression of protein was calculated as the ratio of intensity of the TH, DβH and PNMT bands, respectively, relative to the intensity of β-actin band.

### Transmission electron microscopy (TEM)

Five weeks after surgery, rats were anesthetized with pentobarbital (150mg/kg, i.p.) and perfused transcardially with Ca^2+^- and Mg^2+^-free buffer composed of Dulbecco’s phosphate-buffered saline, 10mM HEPES, 0.2mM ethylene glycol tetraacetic acid, 0.2% bovine serum albumin, 5mM glucose and 9.5mM KCl following the fixative containing 2.5% glutaraldehyde, 2% paraformaldehyde in 0.15M cacodylate buffer as described previously (Pasqua *et al*. 2016). The adrenal gland was removed, sliced into small pieces and postfixed in the same fixative at 4°C overnight. The adrenal slices were stained *en bloc* with 2–3% uranyl acetate for 1h on ice, dehydrated in graded series of ethanol (20–100%) on ice followed by one wash with 100% ethanol and two washes with acetone (15min each) and embedded with Durcupan. Sections (50–60nm thick) were cut on a Leica UCT ultramicrotome, picked up on Formvar and carbon-coated copper grids, and stained with 2% uranyl acetate for 5min and Sato’s lead stain for 1min. Grids were viewed using a JEOL 1200EX II (JEOL, Peabody, MA, USA) TEM and photographed using a Gatan digital camera (Gatan, Pleasanton, CA, USA). Micrographs were randomly taken from three adrenal glands each from sham and HF rats.

### Morphometric analysis

Samples were blinded and two people did measurements randomly from different cells. The line segment tool in ImageJ was used to measure diameters (large dense core vesicle-LDCV, small dense core vesicle-SDCV and small synaptic vesicle-SSV), length (mitochondria) and lumen width (endoplasmic reticulum-ER, Golgi and cristae). The free-hand tool was used to manually trace around the vesicle membrane (LDCV, SDCV and SSV) and mitochondrial outer membrane area and cytoplasm area. For determination of the volume density (%) of vesicles and mitochondria, the sum of the area of the vesicles or mitochondria was divided by the area of the cytoplasm and multiplied by 100 as described previously (Pasqua *et al*. 2016).

### Data presentation and statistical analysis

Data are expressed as mean±s.e.m. Statistical analyses were performed using Student’s *t*-tests, Kolmogorov–Smirnov test as well as one-way ANOVA followed by Dunnett’s *post hoc* test when appropriate. Statistical significance was defined as *P<*0.05. Statistics were computed with the software package, Prism 7 (GraphPad Software, San Diego, CA, USA).

## Results

### General characteristics

**Figure 1:**
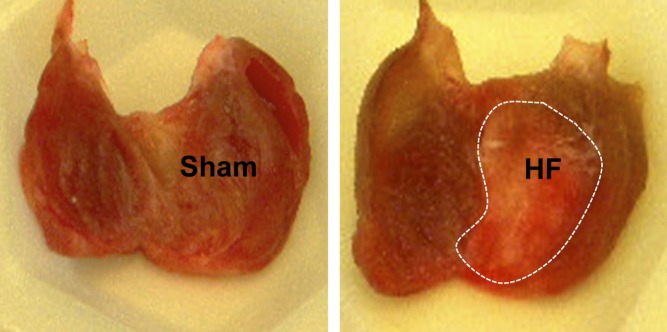
A representative image of the left ventricle from one sham and one HF rat. Circled area indicates infarcted area of the heart in the HF rat. There was no such area in the sham rat. A full colour version of this figure is available at http://dx.doi.org/10.1530/JOE-16-0146.

Table 2 presents general characteristics and left ventricular function parameters among the experimental groups. The body weight and whole heart weight was significantly increased in HF group. Only rats with >30% infarct of the left ventricular wall were included in the study. Sham-operated rats had no visible myocardial damage (Fig. 1). Ejection fraction was significantly lower in HF group. LVEDP was significantly increased in the HF rats compared with sham group. HF rats had significantly ‘lower change in pressure over time’ (+dP/dt and –dP/dt) compared with sham rats suggesting reduced contractility/relaxation. These data confirm that these rats in the HF groups were experiencing cardiac dysfunction as shown in greater detail before (Patel *et al*. 2000, Zhang *et al*. 2001, Kleiber *et al*. 2008).

**Table 2:** The characteristics of sham and HF rats.

**Measures**	**Sham**	**HF**
Body weight (g)	416±14	457±13^*^
Heart weight (g)	1.2±0.1	2.4±0.2^*^
Infarct size (% of epicardial LV)	0	34±2^*^
LVEDP (mmHg)	2±1	24±5^*^
Ejection fraction, %	81±5	51±5^*^
dP/dt, mmHg/s	8549±663	5102±364^*^
−dP/dt, mmHg/s	−6836±346	−4637±298^*^

Values are mean±s.e. *P<0.05 versus sham; *n*=8/group. LV left ventricle, LVEDP left ventricular end-diastolic pressure.

### Increased adrenal weight and adrenal catecholamines in HF rats

**Figure 2:**
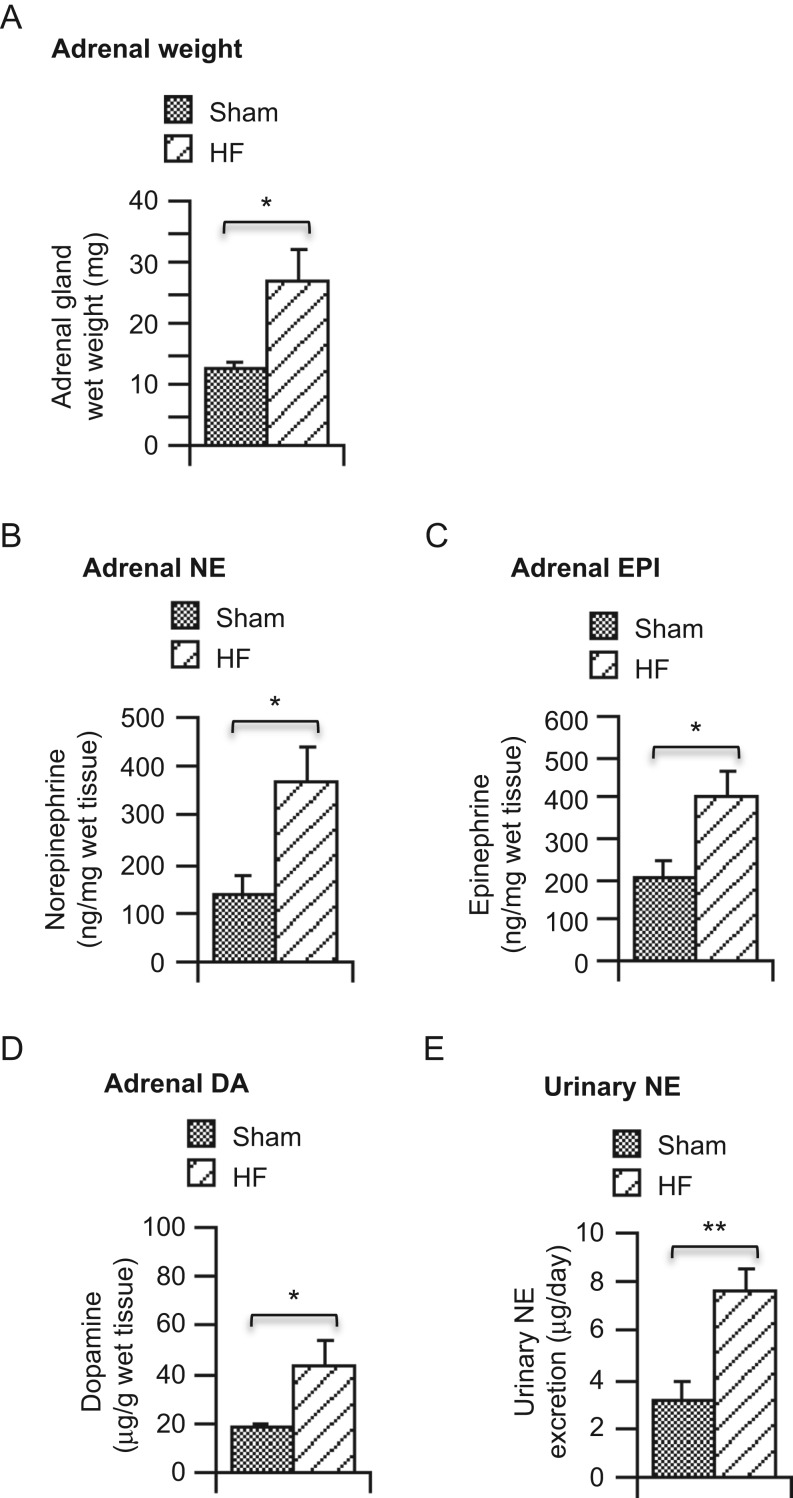
Gravimetry and catecholamine content in sham-operated and HF rats. (A) Weight of the adrenal gland (mg). (B) Adrenal NE content. (C) Adrenal EPI content. (D) Adrenal dopamine (DA) content. (E) Urinary NE level. *P<0.05; **P<0.01.

Consistent with prolonged stress, adrenal glands were heavier in HF rats compared with sham-operated rats (26.7±5.3 HF vs 12.5±1.3mg sham, *P******<*****0.05) (Fig. 2A). Likewise, catecholamine (NE, EPI and DA) contents in the adrenal glands (Fig. 2B–D) were higher in HF rats compared with sham-operated rats. Adrenal NE content was significantly greater in HF rats compared with sham-operated controls (325±35 vs 168±36ng/mg, *P<*0.05). Adrenal EPI was 404±63 HF vs 202±41ng/mg sham (*P<*0.05). Adrenal DA was 46±12 HF vs 18±2μg/g sham (*P<*0.05).

### Urinary norepinephrine excretion measurements

Urinary NE excretion was significantly greater in HF rats compared with sham-operated controls as an index of overall sympathetic activation (7.8±2.3 HF vs 3.2±1.8μg/day sham, *P<*0.05) (Fig. 2E).

### Increased adrenal expression of catecholamine biosynthetic enzymes in HF rats

**Figure 3:**
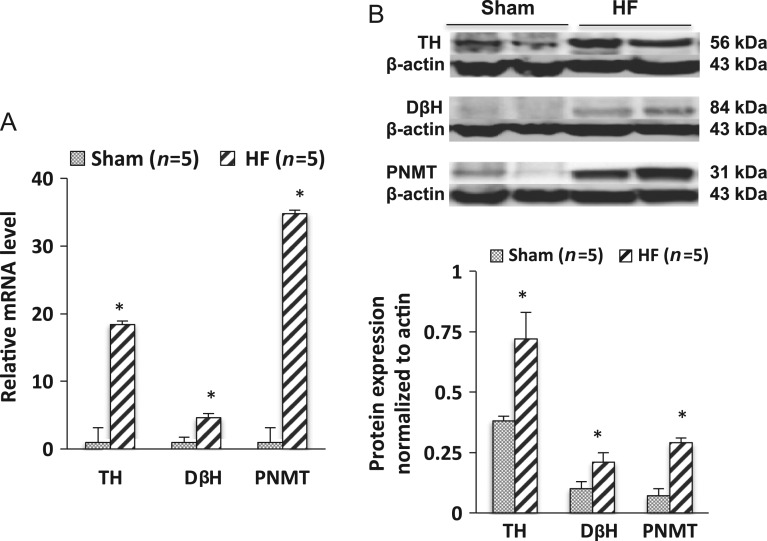
Expression of TH, DβH and PNMT in the adrenal glands of sham and HF rats. (A) TH, DβH and PNMT mRNA expressions relative to GAPDH measured by real-time PCR in sham and HF rats (B) Protein levels of TH, DβH and PNMT in sham and HF rats. *P<0.05; **P<0.01.

Consistent with the increased adrenal catecholamine contents in the adrenal glands in HF rats, the trans-synaptic induction caused significant increase in mRNA and protein expressions of catecholamine biosynthetic enzymes such as TH, DβH, and PNMT in HF rats compared with sham-operated rats (Fig. 3A and B). It is of particular note that there was more than 30-fold increase in the message for PNMT in HF rats.

### Splanchnic nerve terminal and synaptic vesicles

**Figure 4:**
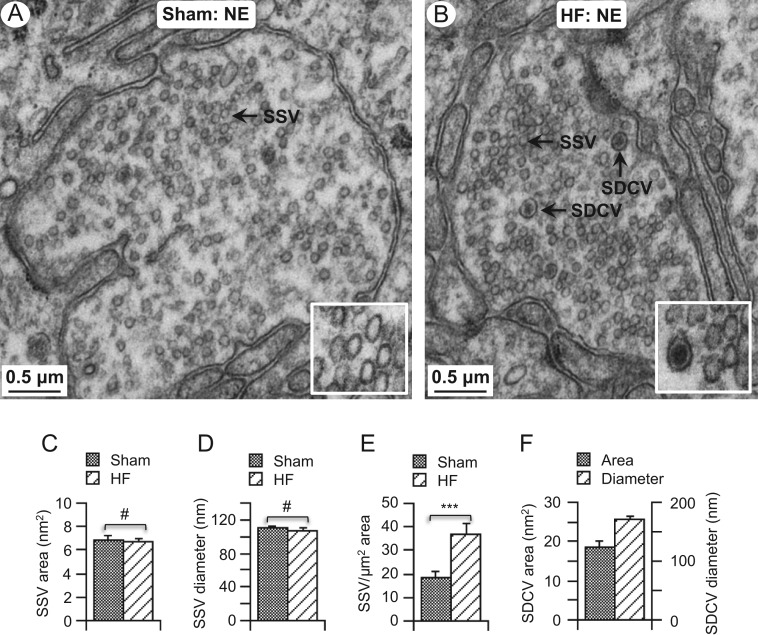
Low-magnification (×2500 original magnification) TEM photographs of the axon terminal. (A) Axon terminal in NE cells of sham-operated rat. (B) Axon terminal in NE cells of HF rat. Morphometric analyses (30 nerve terminals in 30 chromaffin cells from 3 each of the sham-operated and HF rats). (C) SSV area (nm^2^). (D) SSV diameter (nm). (E) SSV per µm^2^ area of the synapse. (F) SDCV area (nm^2^) and SDCV diameter (nm). SDCV, small dense core vesicle; SSV, small synaptic vesicle. ***P<0.001, #: not significant.

The splanchnic–adrenal synapse contains two types of vesicles: clear SSV (presumably cholinergic) and SDCV (presumably peptidergic) (Coupland 1965, Smith & Eiden 2012). In sham-operated rats, we observed SSV in the splanchnic–adrenal synapse (Fig. 4). However, in rats with HF, we found SSV and SDCV in the splanchnic–adrenal synapse. In addition to SDCV, we found significant increased SSV in HF rats.

### Increased volume density, vesicle area and vesicle diameter of NE and EPI granules in HF rats

**Figure 5:**
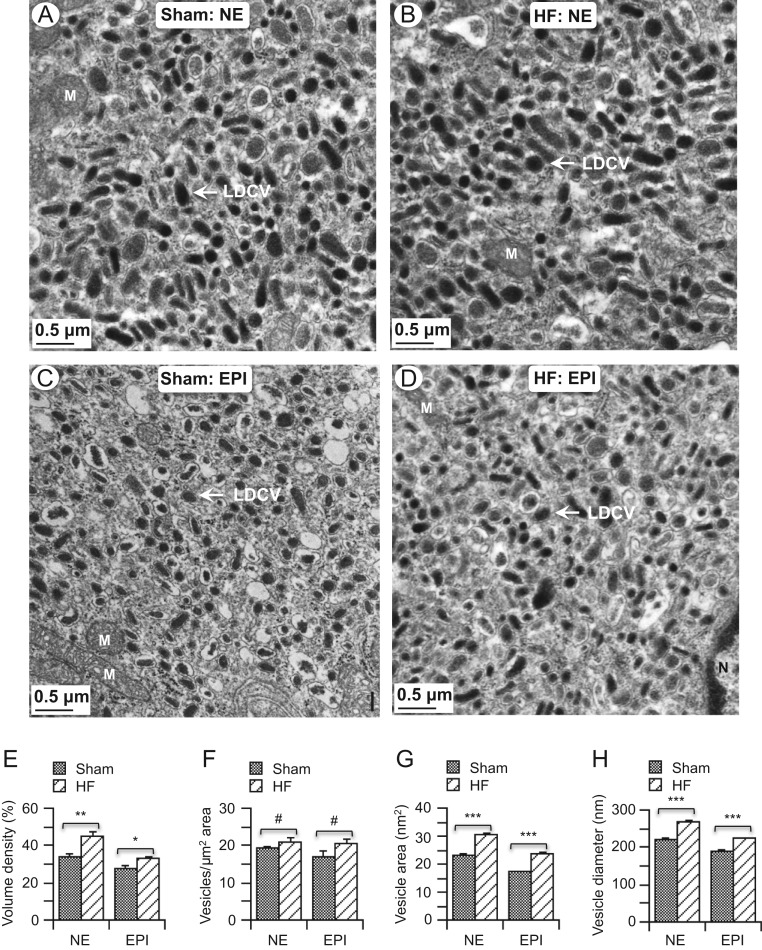
Low-magnification (×2500 original magnification) TEM photographs of the adrenal medulla. (A) Adrenal medulla showing NE cells with LDCV in a sham-operated rat. (B) Adrenal medulla showing NE cells with LDCV in an HF rat. (C) Adrenal medulla showing EPI cells with LDCV in a sham-operated rat. (D) Adrenal medulla showing EPI cells with LDCV in an HF rat. Morphometric analyses (650 vesicles total from 3 each of the sham-operated and HF rats): (E) Volume densities (%) of LDCV. (F) Number of LDCVs per µm^2^ area of the cytoplasm. (G) Vesicle area (nm^2^) of NE-LDCV and EPI-LDCV. (H) LDCV diameter (nm) in NE-LDCV and EPI-LDCV. *P<0.05; **P<0.01; ***P<0.001 and #: not significant.

As shown previously in mouse adrenal medulla (Pasqua *et al*. 2016), the NE-storing vesicles in rats were characterized by typical intensely electron-dense granules, some of which displaying a flattened or tubular appearance (Fig. 5A and B). In contrast, the EPI-storing vesicles were moderately electron dense and smaller in size (Fig. 5C and D). Although there were no changes in numerical density of the LDCV in chromaffin cells between sham-operated and HF rats (Fig. 5F), HF rats displayed increased LDCV volume density (Fig. 5E) coupled with increased LDCV area (Fig. 5G) and LDCV diameter (Fig. 5H) compared with sham-operated rats.

### Relative frequency distribution of NE-LDCV and EPI-LDCV in sham and HF rats

**Figure 6:**
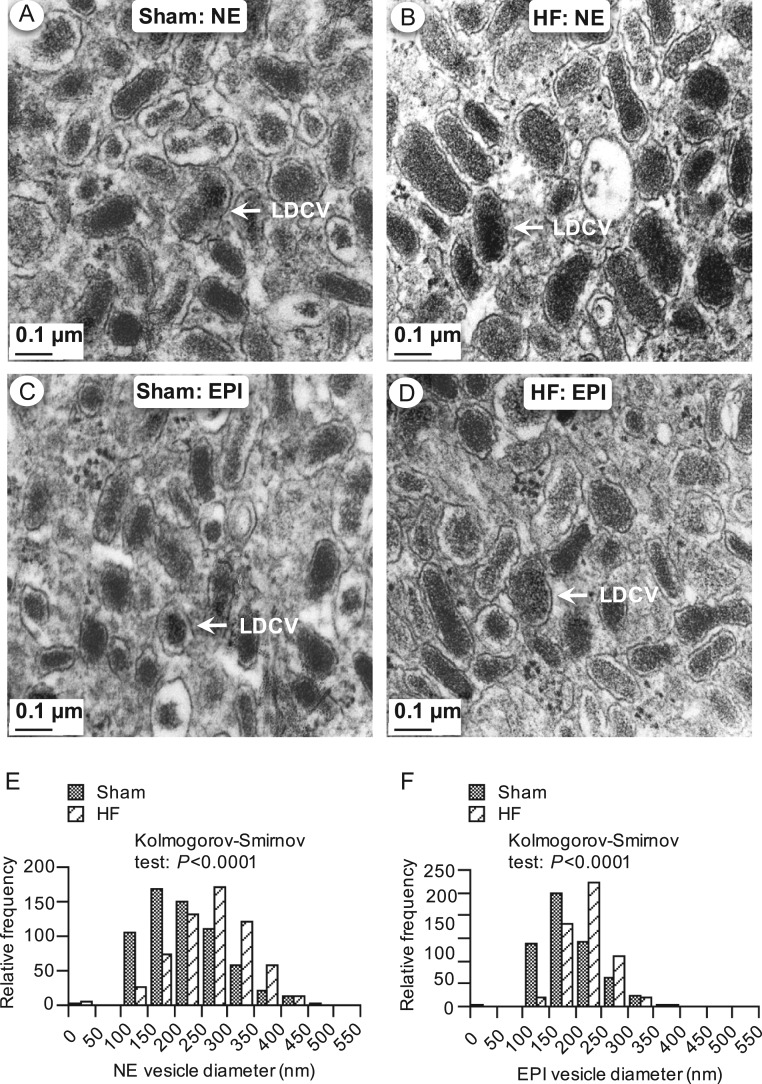
High magnification (×15,000 original magnification) TEM photographs of the adrenal medulla. (A) Adrenal medulla showing NE cells with LDCV in a sham-operated rat. (B) Adrenal medulla showing NE cells with LDCV in an HF rat. (C) Adrenal medulla showing EPI cells with LDCV in a sham-operated rat. (D) Adrenal medulla showing EPI cells with LDCV in an HF rat. Morphometric analyses were carried out in 650 vesicles total from 3 each of the sham-operated and HF rats. Relative frequency distribution of NE-LDCV diameter (E) and EPI-LDCV diameter (F) are shown. Note highly significant (*P*<0.0001) right shift in the distribution of NE and EPI vesicular diameter with heart failure.

While 100–250nm LDCV diameters of NE-storing cells predominate in sham-operated rats, the preponderant LDCV diameters in HF rats ranged from 250 to 400nm (Fig. 6A, B and E). Kolmogorov–Smirnov test revealed highly significant right shift in the distribution of vesicular diameter with heart failure (Kolmogorov–Smirnov D=0.29; *P<*0.0001). Similarly, LDCV diameters in EPI-storing vesicles were higher in HF rats (200–300nm) compared with sham-operated rats (Fig. 6C, D and F). Like NE vesicles, there was a significant right-ward shift in the distribution of EPI vesicular diameters in rats with heart failure as evaluated by Kolmogorov–Smirnov test (Kolmogorov–Smirnov D=0.36; *P<*0.0001).

### Rough endoplasmic reticulum (RER) and Golgi complex (GC)

**Figure 7:**
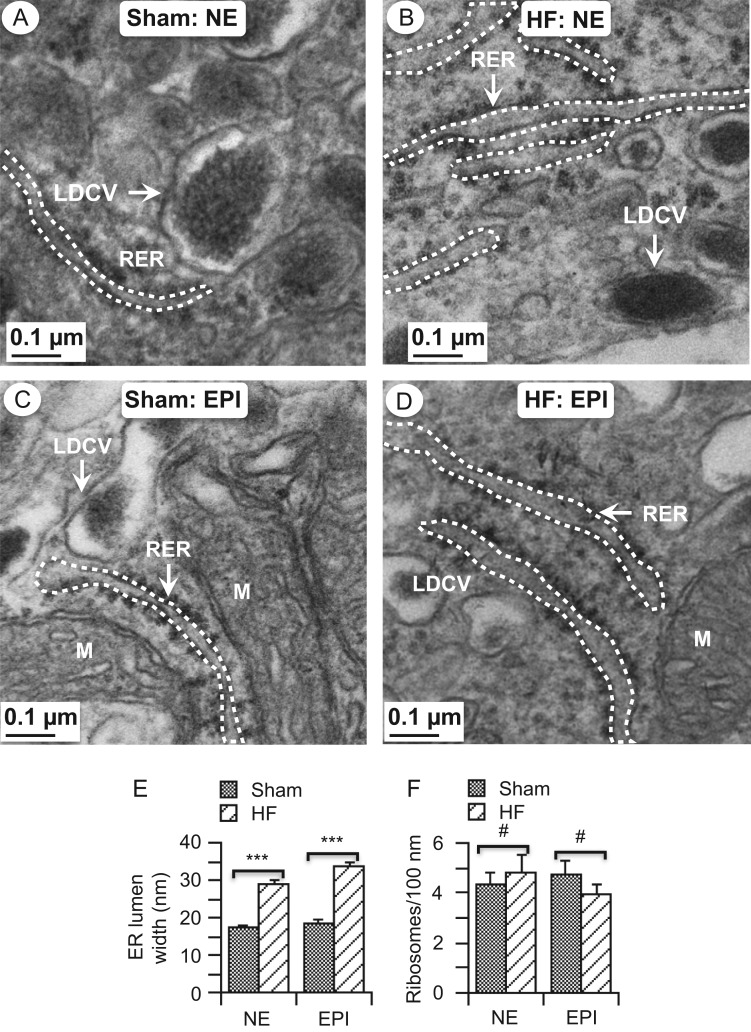
High magnification (×15,000 original magnification) TEM photographs of rough endoplasmic reticulum (RER). (A) NE cells showing RER and LDCV in a sham-operated rat. (B) NE cells showing RER (dilated) and LDCV in an HF rat. (C) EPI cells showing RER and LDCV in a sham-operated rat. (D) EPI cells showing RER (dilated) and LDCV in an HF rat. Morphometric analyses (2 RER per chromaffin cell for a total of 60 RER in 30 cells from 3 each of the sham-operated and HF rats). (E) Average ER lumen width (nm). (F) Average ribosomes per 100nm length of ER. ***P<0.001; # not significant.

ER regulates the synthesis, folding and maturation of secreted and transmembrane proteins and the storage of Ca^2+^, lipid biosynthesis and redox homeostasis (Oakes & Papa 2015). Since HF rats suffer from prolonged stress, the adrenal medulla synthesized and secreted more catecholamines compared with sham-operated rats to cope with the stress situation. Increased catecholamine synthesis requires increased synthesis of catecholamine biosynthetic enzymes. Consistent with the above, HF rats displayed increased RER lumen width in both NE and EPI storing cells compared with sham-operated rats (Fig. 7A–E). Surprisingly, ribosomal numerical density did not change between HF and sham-operated rats (Fig. 7F).

**Figure 8:**
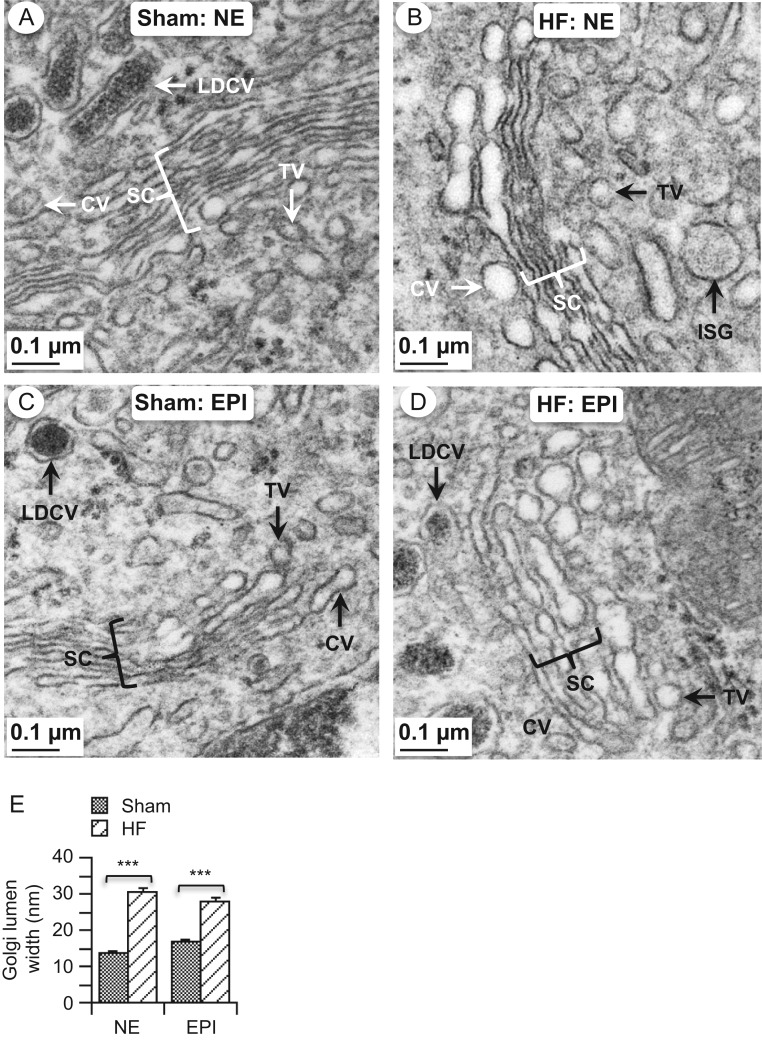
High-magnification (×15,000 original magnification) TEM photographs of Golgi complex (GC). (A) NE cells showing GC and LDCV in a sham-operated rat. (B) NE cells showing GC (dilated) and ISG in an HF rat. (C) EPI cells showing GC and LDCV in a sham-operated rat. (D) EPI cells showing GC (dilated) and LDCV in an HF rat. Morphometric analyses (1 GC per chromaffin cell for a total of 60 GC from 3 each of the sham-operated and HF rats). (E) Average GC lumen width. CV, cis-Golgi vesicles; ISG, immature secretory granule; TV, trans-Golgi vesicle; SC, stacked-Golgi cisternae. ***P<0.001.

GC is the site of processing, packaging and sorting of proteins and lipids *en route* from the ER to the plasma membrane and other destinations (Sengupta & Linstedt 2011). Increased catecholamine synthesis in HF rats would require formation of more vesicles for catecholamine storage. This increased workload has resulted in dilation of GC lumen in HF rats compared with sham-operated rats (Fig. 8A–E).

### Mitochondrial lengths, volume and numerical densities and cristae volume density

**Figure 9:**
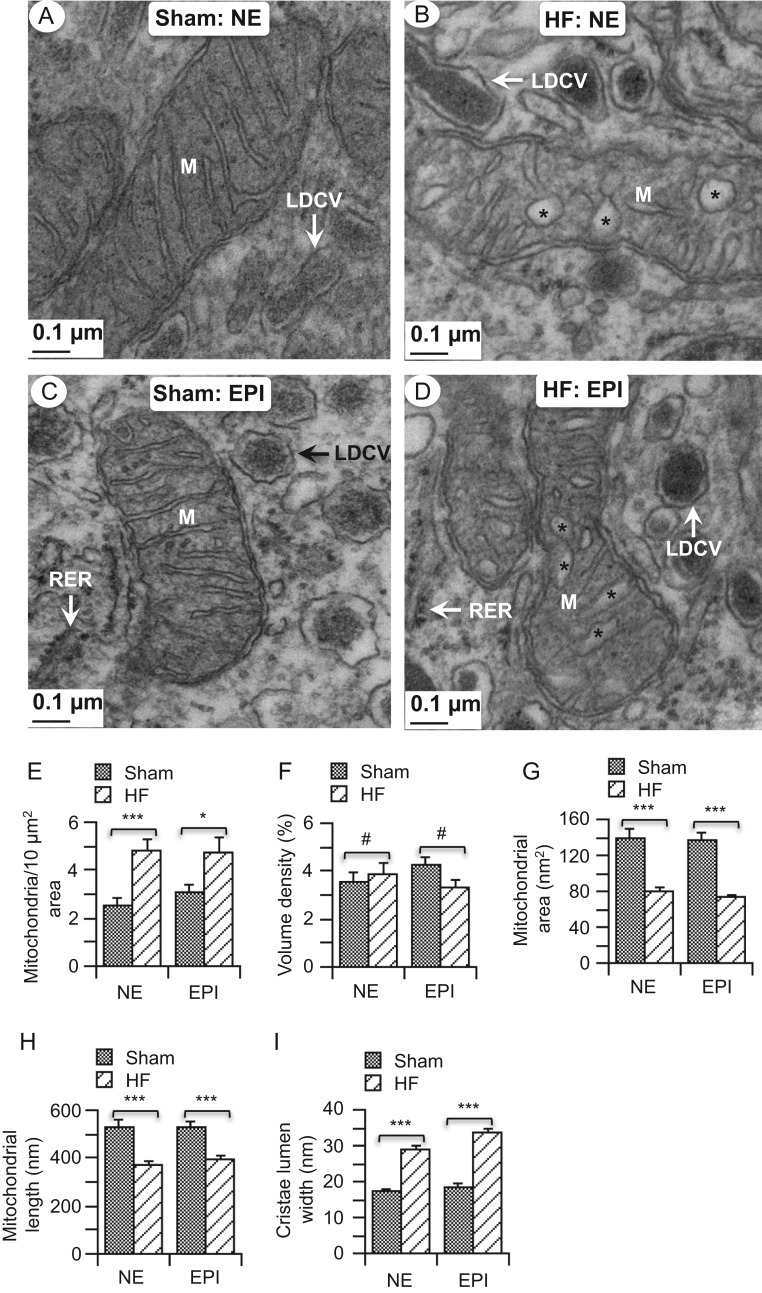
High-magnification (x15,000 original magnification) TEM photographs of mitochondria. (A) NE cells showing tubulo-lamellar mitochondria and LDCVs in a sham-operated rat. (B) NE cells showing a tubulo-vesicular mitochondrion and LDCVs in an HF rat. (C) EPI cells showing a tubulo-lamellar mitochondrion and LDCVs in a sham-operated rat. (D) EPI cells showing tubulo-vesicular mitochondria and LDCVs in an HF rat. Morphometric analyses (60 mitochondria total from 3 each of the sham-operated and HF rats): (E) Average mitochondria numbers per 10µm^2^ area of the cytoplasm. (F) Average mitochondrial volume density (%). (G) Average mitochondrial area (nm^2^). (H) Average mitochondrial length (nm). (I) Average cristae lumen width (nm). *P<0.05; ***P<0.001; # not significant.

Mitochondria regulates the life and death of a cell by providing the enzymes and structural framework for generation of adenosine triphosphate (ATP), which serves as the energy currency of the cell (Scheffler 2008). Mitochondria in NE- and EPI-storing sham-operated rats showed the typical tubulo-lamellar appearance (Fig. 9A and C). In HF rats, mitochondrial cristae were dilated and looked like tubulo-vesicular mitochondria (Fig. 9B and D). Since increased ATP synthesis is required to counteract the prolonged stress, HF rats responded by increased biogenesis of mitochondria as shown by increased mitochondrial numerical density (Fig. 9E), which is accompanied by decreased mitochondrial area (Fig. 9G) and length (Fig. 9H). Despite increased mitochondrial numerical density in HF rats, there was no change in mitochondrial volume density (Fig. 9F) because of the smaller mitochondrial area (Fig. 9E–G). In addition to increased biogenesis of mitochondria, HF rats showed increased cristae lumen width to meet the increased energy demand in HF rats (Fig. 9I).

### Changes in glycogen granules

**Figure 10:**
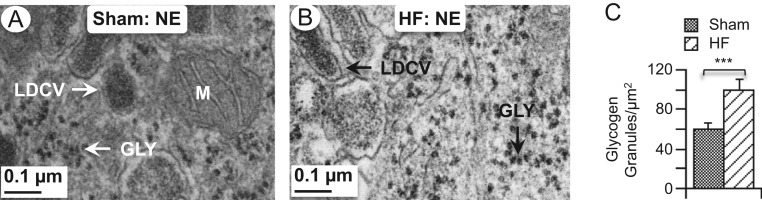
High-magnification (×15,000 original magnification) TEM photographs showing glycogen granules. (A) NE cells showing glycogen granules, LDCVs and mitochondrion in a sham-operated rat. (B) NE cells showing glycogen granules and LDCVs in an HF rat. (C) Morphometric data (60 cells total from 3 each of the sham-operated and HF rats) showing number of glycogen granules per µm^2^ of the cytoplasm. ***P<0.001.

At the ultrastructural level, glycogen appears as roughly circular granules from 150 to 400Å in diameter (Revel *et al*. 1960). Nutrient (high-fat diet) and hyperadrenergic (increased plasma catecholamines) stress appear to increase storage of glycogen for steady glucose supply to meet the high-energy demand (Pasqua *et al*. 2016). Consistent with the above stressors, we found an increased number of glycogen granules in HF rats compared with sham-operated rats (Fig. 10A–C).

## Discussion

This study provides novel electron micrographic evidence to characterize the fundamental changes in the adrenomedullary chromaffin cells related to the status of the sympathetic nervous system activation during the HF condition. First, conscious undisturbed rats with HF display an enhanced adrenal medullary activity in rats with HF. Second, SDCV (possibly containing pituitary adenylate cylase-activating peptide (PACAP) and vasoactive intestinal peptide (VIP)) in splanchnic–adrenal synapse of HF rats aids in maintaining long-term catecholamine secretion using secretory mechanisms different from that of acetylcholine. Third, increased ER and Golgi lumen width in HF rats are consistent with the demand of increased catecholamine synthesis and release. Fifth, more mitochondria with dilated cristae suggest increased ATP production, which is required for increased LDCV priming and subsequent exocytosis in rats with HF. Sixth, increased glycogen content in HF rats is indicative of a metabolic switch to accommodate the HF-induced prolonged stress. Taken together, these data provide a comprehensive characterization of the adaptive changes indicative of increased machinery for enhanced adrenal activity in the adrenal medullary chromaffin cells to provide chronic activation of the adrenal medulla during the chronic HF condition.

The adrenal medulla is innervated by preganglionic sympathetic neurons that are located in the intermediolateral cell column of the thoracic spinal cord and extend out to the adrenal medulla through thoracic splanchnic nerves (Wakade 1988, Mahata *et al*. 2000). The splanchnic–adrenal synapse contains both cholinergic (such as acetylcholine in SSV) and peptidergic (such as PACAP, VIP and secretin in SDCV) neurotransmitters (Wakade 1988, Guo & Wakade 1994, Taupenot *et al*. 1998, Mahata *et al*. 2000). While acetylcholine stimulates the secretion of NE and EPI, PACAP and VIP predominantly stimulate the secretion of EPI (Guo & Wakade 1994). The low-frequency splanchnic nerve firing (mainly by acetylcholine) causes catecholamine secretion from the adrenal medulla at a modest rate to maintain basal functioning. In contrast, the high-frequency firing (mainly PACAP and also acetylcholine) associated with emergency (stress) responses induce the adrenal medulla to secrete catecholamines at an elevated rate to combat with the perceived stress situation (Smith & Eiden 2012, Stroth *et al*. 2013).

Consistent with the above findings and hypothesis, we found SDCV (possibly containing PACAP and VIP) in splanchnic–adrenal synapse of HF rats. Existing literature suggest that PACAP maintains long-term catecholamine secretion using secretory mechanisms different from that of acetylcholine (rapidly desensitizing) and via induction of the catecholamine biosynthetic enzymes such as TH, DβH and PNMT (Taupenot *et al*. 1999, Smith & Eiden 2012, Stroth *et al*. 2013). Consistent with this idea in fact, increased mRNA levels of TH and PNMT in HF have been reported previously in pig (Tomaszek *et al*. 2015). Coherent with this, in fact, the trans-synaptic induction caused a dramatic and significant increase in TH, DβH and PNMT mRNA expression as well as protein in rats with HF. This upregulation was likely triggered by enhanced sympathetic tone commonly reported in HF.

Catecholamine secretion from adrenal medullary chromaffin cells is influenced by both cholinergic and peptidergic stimuli conducted by preganglionic sympathetic neurons that are located in the intermediolateral cell column of the thoracic spinal cord and extend to the adrenal medulla through thoracic splanchnic nerves (Mirkin 1961). The splanchnic–adrenal synapse contains two types of vesicles: clear SSV (presumably cholinergic) and SDCV (presumably peptidergic) (Coupland 1965, Smith & Eiden 2012). Cholinergic (acetylcholine) and peptidergic (such as PACAP and VIP) transmitters are preferentially released from this splanchnic–adrenal synapse: acetylcholine from SSV is released during low-frequency firing to release catecholamines at a modest rate for basal functioning; PACAP is released from SDCV along with acetylcholine during high-frequency firing upon stress-induced heightened sympathetic activation and releases catecholamines from LDCV at a much higher rate to accommodate the stress situation (Smith & Eiden 2012). In this study, we found normal levels of SSV in the splanchnic–adrenal synapse in sham-operated rats, whereas in rats with HF, we found a significant increase in SSV and SDCV in the splanchnic–adrenal synapse. These data indicate that the preganglionic sympathetic innervation to the adrenal medulla is primed for overactivation and sustained activation in rats with HF. It is likely that the increased SSV and SDCV aid in the chronic long-term activation of the adrenal medullary chromaffin cells in the chronic HF condition.

The body responds to stress by activating two hormonal systems: (1) the hypothalamus–pituitary–adrenal (HPA) axis, which causes the release of corticotrophin-releasing hormone (CRH) from the hypothalamus, followed by adrenocorticotrophic hormone (ACTH) from the anterior pituitary in response to CRH and finally cortisol (human) or corticosterone (rodents) from the adrenal cortex in response to ACTH and (2) brainstem catecholaminergic neurons and spinal cord efferent activates the sympathetic nervous system and adrenal medulla to secrete NE (from the sympathetic nervous system and adrenal medulla) and EPI (from the adrenal medulla) (Tank & Lee Wong 2015). As shown previously in adrenal medulla, the NE-storing vesicles are typically characterized by intense electron-dense granules, some of which displaying a flattened or tubular appearance. In contrast, the EPI-storing vesicles are moderately electron dense and smaller in size (Pasqua *et al*. 2016). This distinction allowed us to quantify the changes in these two distinct types of chromaffin cells in rats with HF.

The stress-induced release of NE and EPI varies dramatically in response to various stressors. For example, insulin-induced hypoglycemia or 2-deoxyglucose-induced glucoprivation causes profound release of EPI (by 10- to 30-fold) compared with a modest release of NE (by twofold) (Blandini *et al*. 1995, Elman *et al*. 2001). Surgical stress also causes profound release of EPI (Mannelli *et al*. 1982). In contrast, cold stress causes more release of NE than EPI (Johnson *et al*. 1977). Differential release of NE and EPI in response to different stressors has also been documented in animal studies (Pacak *et al*. 1998). In HF, there is general overactivation of the sympathetic nervous system. In mice, HF causes release of more NE (by 7.5-fold) and EPI (by 2.8-fold) (Kamal *et al*. 2014). This is indicative of an elevated overall sympathoexcitation to the general circulation during HF. It is of interest to note that previously we have shown that CgA-deficient *Chga*-KO mice also suffer from persistent systematic stress due to increased secretion of catecholamines compared with WT mice, which leads to hypertension and also demonstrates majority of the morphological changes that are observed in the hypersympathetic state observed in the HF condition in this study (Pasqua *et al*. 2016). This suggests an intriguing overarching global conclusion that hyperactivated sympathetic state, regardless of its initial cause or origin can demonstrate similar morphological changes in the activated adrenal glands dictating catecholamine synthesis, storage and release.

It should be noted that there is strong evidence for a regionally heterogeneous pattern of sympathetic activation that appears to be targeted to specific tissues in experimental HF. Increased NE spillover from heart and kidney (but not lung) (Hasking *et al*. 1986, Eisenhofer *et al*. 1996, Rundqvist *et al*. 1997) and increased burst number in postganglionic sympathetic efferent to the vasculature of skeletal muscle (Ferguson *et al*. 1990) have been described in clinical HF. We have previously shown an increased turnover of NE in the heart, kidney and skeletal muscle, but not in spleen and liver in rats with HF (Patel *et al*. 2000). NE turnover can be influenced by any event that affects synthesis, release, uptake or breakdown of NE at the noradrenergic nerve terminal. For example, increased circulating or local angiotensin II levels in HF could stimulate NE release from peripheral sympathetic nerve endings (Clemson *et al*. 1994, Driessen & Starke 1994), thus promoting increased NE turnover. Noradrenergic processes at the level of nerve terminal that are independent of neural activity appear to be unaltered in rats with HF, which also implicates centrally mediated neural mechanisms in causing the regional increases in NE turnover. In this study, the changes in the adrenal medulla appear to be dictated by an overactive state during HF.

This study shows a significant activation of the adrenal medulla of rats with HF. The close parallel between the results of these studies and the clinical literature strengthens the contention that sympathetic activation of some (but not all) tissues accompanies HF. Our findings in rats are also consistent with the findings in mice, where more plasma catecholamines are compensated by more resynthesis (as evidenced by more NE and EPI in the adrenal gland) (Kamal *et al*. 2014). We found increased volume density, LDCV area and LDCV diameter for both NE-storing cells as well as EPI-storing vesicles in HF rats compared with sham rats. These results indicate the increased machinery for enhanced adrenal activity in rats with HF.

The findings of increased ER (for synthesis of more catecholamine biosynthetic enzymes and SNARE proteins involved in exocytosis) and Golgi lumen (for formation of more catecholamine storage vesicles) width in HF rats are consistent with the demand of increased catecholamine synthesis and release. In the chronic HF condition, elevated plasma levels of catecholamines would require additional synthesis and release that perhaps are maintained by this increased ER in the chromaffin cells.

 The peroxisome proliferator-activated receptor-γ (PPARγ) coactivator 1α (PGC-1α) is a major regulator of exercise-induced phenotypic adaptation and substrate utilization. A modest (~25%) upregulation of PGC-1α improves mitochondrial biogenesis and fatty acid oxidation in healthy and insulin-resistant skeletal muscle. It has been reported that PGC-1α is involved in the coordinated regulation of nuclear- and mitochondrial-encoded genes required for contractile and metabolic adaptations in skeletal muscle (Puigserver & Spiegelman 2003, Scarpulla 2008, Wende *et al*. 2007). Of note, PGC-1α expression *per se* is also regulated by PGC-1α via its interaction with MEF2 (Handschin *et al*. 2003). Based on the above literature, we believe that increased mitochondrial biogenesis in NE and EPI cells in HF rats is due to increased expression of PGC-1α (Karamanlidis *et al*. 2010, Ahuja *et al*. 2013). Decreased mitochondrial area and diameter without any change in mitochondrial volume density are indicative of more but smaller mitochondria in HF rats. Cellular respiration takes place in the mitochondrial cristae where biochemical energy from nutrients is converted into ATP, the energy currency of the cell. In response to increased energy demand, cristae increase its surface area by increasing cristae lumen width to produce more ATP. Thus, dilated cristae in HF rats are consistent with increased ATP production required for increased LDCV priming and consequent exocytosis (Rettig & Neher 2002).

Heart switches from less energetically efficient fatty acids substrate to more energetically efficient carbohydrate substrate for ATP production to protect the myocardium under ischemic conditions (Neubauer 2007, Aragones *et al*. 2009). Similar metabolic switch for ATP production also takes place in cancer cells (Vander Heiden *et al*. 2009). Therefore, increased glycogen content in the chromaffin cells of the adrenal medulla from the rats with HF may indicate a metabolic switch from fatty acid to carbohydrate metabolism to counteract HF-induced prolonged synthesis and release of catecholamines by the chromaffin cells.

In summary, the adrenal medulla is activated during the chronic HF condition. These electron micrographic observation indicates that the chronic HF condition provokes increased basal SDCV (possibly containing PACAP and VIP) in splanchnic–adrenal synapse of HF rats promoting toward the maintenance of long-term catecholamine secretion, increased ER and Golgi lumen width to accommodate the enhanced demand for increased catecholamine synthesis and release, and more mitochondria with dilated cristae and glycogen to adapt to the increased energy demand for the increased biogenesis and exocytosis of catecholamines from the adrenal medulla. Thus, although centrally mediated sympathetic activation accompanies chronic HF, the results in this study demonstrates heterogeneity of adaptive changes within the adrenal chromaffin cells that likely contribute to the functional consequences of chronic elevations in plasma catecholamines in pathophysiological state of chronic HF.

## Declaration of interest

The authors declare that there is no conflict of interest that could be perceived as prejudicing the impartiality of the research reported.

## Funding

This work was supported by the National Institutes of Health grants R56 HL124104 and P01 HL62222.
